# Time trends and social inequalities in infant and young child feeding practices: national estimates from Brazil’s Food and Nutrition Surveillance System, 2008–2019

**DOI:** 10.1017/S1368980023001039

**Published:** 2023-09

**Authors:** Giesy Ribeiro de Souza, Rita de Cássia Ribeiro-Silva, Mariana Santos Felisbino-Mendes, Natanael de Jesus Silva, Rafaella da Costa Santin de Andrade, Jéssica Pedroso, Ana Maria Spaniol, Gisele Ane Bortolini, Eduardo Augusto Fernandes Nilson, Sara Araújo da Silva, Bárbara Hatzlhoffer Lourenço, Aline dos Santos Rocha, Ila Rocha Falcão, Maria Yury Travassos Ichihara, Dayana Rodrigues Farias, Maurício Lima Barreto

**Affiliations:** 1 Centre for Data and Knowledge Integration for Health, Gonçalo Moniz Institute, Oswaldo Cruz Foundation, Salvador, Bahia 41745-715, Brazil; 2 Institute of Collective Health, Federal University of Bahia, Salvador, Bahia, Brazil; 3 School of Nutrition, Federal University of Bahia, Salvador, Bahia, Brazil; 4 School of Nursing, Department of Mother-Child Nursing and Public Health, Federal University of Minas Gerais, Belo Horizonte, Minas Gerais, Brazil; 5 Barcelona Institute for Global Health, Hospital Clinic, Universitat de Barcelona, Barcelona, Spain; 6 General Coordination of Food and Nutrition, Ministry of Health, Federal District, Brasília, Brazil; 7 Food, Nutrition and Culture Program (PALIN), Brasília Regional Management, Oswaldo Cruz Foundation, Federal District, Brasília, Brazil; 8 Department of Nutrition, School of Public Health, University of São Paulo, São Paulo, SP, Brazil; 9 Nutritional Epidemiology Observatory, Josué de Castro Institute of Nutrition, Federal University of Rio de Janeiro, Rio de Janeiro, Brazil

**Keywords:** Breast-feeding, complementary feeding, time-series studies, Brazilian deprivation index, child nutrition

## Abstract

**Objective:**

To describe the time trends and socio-economic inequalities in infant and young child feeding practices in accordance with the Brazilian deprivation index (BDI).

**Design:**

This time-series study analysed the prevalence of multiple breast-feeding and complementary feeding indicators based on data from the Brazilian Food and Nutrition Surveillance System, 2008–2019. Prais–Winsten regression models were used to analyse time trends. Annual percent change (APC) and 95 % CI were calculated.

**Setting:**

Primary health care services, Brazil.

**Participants:**

Totally, 911 735 Brazilian children under 2 years old.

**Results:**

Breast-feeding and complementary feeding practices differed between the extreme BDI quintiles. Overall, the results were more favourable in the municipalities with less deprivation (Q1). Improvements in some complementary feeding indicators were observed over time and evidenced such disparities: minimum dietary diversity (Q1: Δ 47·8–52·2 %, APC + 1·44, *P* = 0·006), minimum acceptable diet (Q1: Δ 34·5–40·5 %, APC + 5·17, *P* = 0·004) and consumption of meat and/or eggs (Q1: Δ 59·7–80·3 %, APC + 6·26, *P* < 0·001; and Q5: Δ 65·7–70·7 %, APC + 2·20, *P* = 0·041). Stable trends in exclusive breast-feeding and decreasing trends in the consumption of sweetened drinks and ultra-processed foods were also observed regardless the level of the deprivation.

**Conclusions:**

Improvements in some complementary food indicators were observed over time. However, the improvements were not equally distributed among the BDI quintiles, with children from the municipalities with less deprivation benefiting the most.

Breast-feeding and healthy complementary feeding are essential practices for the health promotion in children under 2 years of age^(1)^. Evidence shows that the beneficial effects of these practices can extend into adulthood^(2)^. On the other hand, inadequate feeding practices in early life can increase the risk of morbidity and mortality, as they contribute to the adherence to poor dietary practices in other life stages as well as the development of non-communicable chronic diseases^(2–5)^.

The WHO began to recommend that babies be exclusively breastfed in 1990^(6)^, and since 2001 has stated that the optimal duration is 6 months (180 d)^(7)^. Overall, the increase in breastfeeding prevalence has been reported in various studies worldwide, especially for exclusive breastfeeding in children under 6 months^(8–10)^. In 2019, the UNICEF stated that exclusive breastfeeding had increased from 35 % in 2005 to 42 % in 2018 in eighty low- and middle-income countries^(11)^. In Brazil, the latest national survey on breastfeeding in 2019 and 2020 showed that the recommendation of the WHO has not been met, as 45·8 % of children received breast milk in the country^(12)^. This observation is particularly apparent for exclusive breastfeeding and continued breastfeeding in the second year of life. The WHO goal for 2030 is that at least 70 % of children under the age of 6 months are breastfed exclusively^(13)^.

The WHO and UNICEF recognise the considerable impact of inadequate feeding practice on poor early nutrition^(14)^. Therefore, they endorse exclusive breastfeeding in the first 6 months of life, followed by the safe introduction of complementary foods, with continued breastfeeding until 2 years of age^(15)^. In order to guarantee healthy complementary feeding practices, it is important that children consume foods with the appropriate consistency, diversity and frequency for their age. These conditions are represented in some of the main indicators of feeding practices for infants and young children recommended by the WHO and adopted by the Ministry of Health in Brazil^(6,16,17)^. These are (a) Introduction of solid, semi-solid or soft foods, (b) Minimum dietary diversity, (c) Minimum meal frequency, (d) Minimum acceptable diet and (e) Consumption of Fe-rich or Fe-fortified foods.

In contexts of socio-economic inequalities, the use of these indicators could be crucial, considering the immense threat that these extreme conditions may represent to ideal feeding practices^(18–22)^. This inability to provide adequate nutrition to small children (6–23·9 months) has a direct impact on their micronutrient status and growth^(15)^. In Brazil, the monitoring of nutritional status is part of the Food and Nutrition Surveillance (VAN), provided for in the law that created the Unified Health System (SUS), which consists of the continuous description of the food and nutrition conditions of the Brazilian population^(23)^. Despite the actions taken by the Ministry of Health regarding the expansion of Food and Nutrition Surveillance (VAN), the increase in the population at levels of serious poverty, high inflation on food prices and expenditure restraint on essential public policies, such as education and health, have severely compromised the food and nutrition security of Brazilian families, especially women and children^(23–25)^.

As with many developing countries, overcoming inadequate feeding practices remains a challenge for Brazil. According to the National Study of Infant Food and Nutrition, the prevalence of minimum food frequency was 39.2 %, and the prevalence of minimum dietary diversity was 57.1 % among Brazilian children aged 6–23 months^(12)^. This is a worrisome condition, as adequate and healthy food is a fundamental right of every child and a duty of the Brazilian State^(26)^. We were motivated to explore the theme because of the lack of a systematic assessment of feeding indicators in infants and children under age 2 in Brazil, along with the relevance of assessing feeding trends over time to understand what changes have occurred, and to subsidise the planning of strategies to promote healthier diets for Brazilian children. Thus, the aim of this study is to describe the temporal trends in the prevalence of breastfeeding and complementary feeding indicators among children assisted by primary health care services in the Sistema Único de Saúde (SUS) between 2008 and 2019, according to the Brazilian deprivation index (BDI).

## Methods

### Study design and population

This is a time series study. Data were obtained from the Food and Nutrition Surveillance System (SISVAN) which includes data on food consumption of children under 2 years of age who assisted SUS primary care services between 2008 and 2019.

**Fig. 1:**
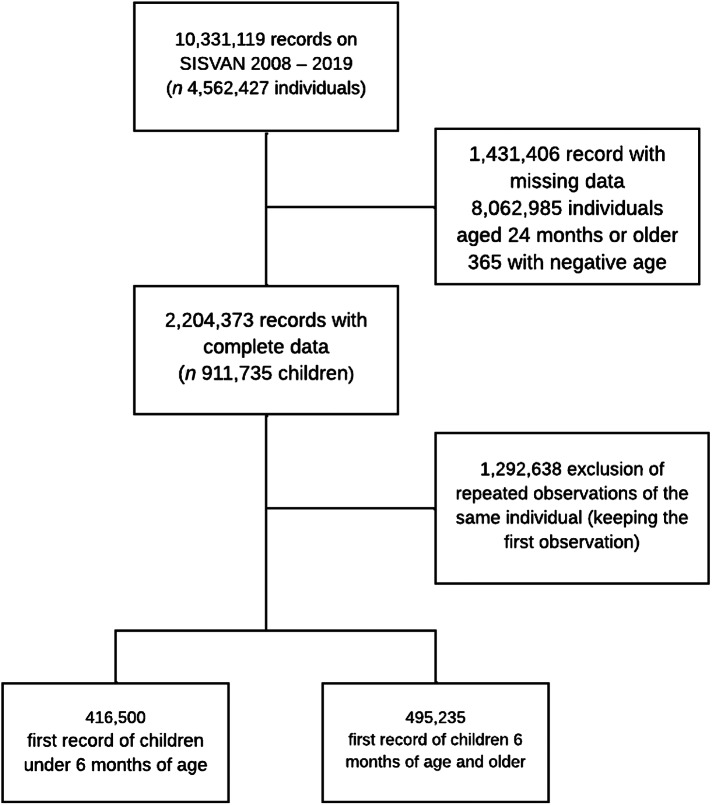
Selection of the study population. Food and Nutritional Surveillance System, 2008–2019

Data access, processing and analysis were conducted at the Centre for Data and Knowledge Integration for Health (CIDACS) of the Oswaldo Cruz Foundation (FIOCRUZ)^(27)^. All children under 2 years of age with at least one entry record and a measure of food consumption were included. The most recent record per year of each assisted child was considered to estimate the prevalence of breastfeeding and complementary feeding indicators (Fig. 1).

### Indicators of infant and young child feeding practices

Food consumption records from SISVAN were used to calculate the following child feeding practice indicators: exclusive breastfeeding; mixed breastfeeding; continued breastfeeding; introduction of solid, semi-solid or soft foods (ISSSF); minimum meal frequency; minimum dietary diversity (MDD); minimum acceptable diet (MAD); consumption of meat and/or eggs; consumption of sweetened beverages; consumption of ultra-processed foods (UPF); consumption of Fe-rich foods; consumption of foods rich in vitamin A and zero consumption of fruit and vegetables^(6,16)^. Food consumption was evaluated using a standardised form with specific questions to qualitatively measure feeding practices the day before^(16)^. The summary of the indicators is provided in Chart 1. The forms used in SUS primary health care services to obtain food consumption information were based on a document on indicators for assessing infant and young child feeding practices published by the WHO and revised by the Brazilian Ministry of Health in late 2014, with the aim of simplifying data collection and analysis of the information^(16,17)^. Accordingly, we opted to analyse the indicators of child feeding practices in two periods of time (2008–2014 and 2015–2019). For questions which did not change over time, or were equivalent, the analysis was conducted over the total study period (2008–2019). To evaluate the percentage of children that are monitored by SISVAN per year, the coverage was calculated from the ratio between the number of individuals with food consumption records and the total population under 2 years old, multiplied by 100.

**Chart 1:** Indicators in infant and young child feeding practices. Food and Nutrition Surveillance System (SISVAN)

Indicators	Description	Formula
Exclusive breast-feeding up to 6 months*	Percentage of children aged up to 5 months and 29 d who exclusively received breast milk on the day prior to the evaluation	Children up to 5 months and 29 d who received breast milk Total of children up to 5 months and 29 d
Mixed breastfeeding**	Percentage of children aged up to 5 months and 29 d who received formula and/or animal milk and breast milk on the day prior to the evaluation.	Children aged up to 5 months and 29 d who received breast milk and formula and/or animal milk Total of children aged up to 5 months and 29 d
Continued breastfeeding	Percentage of children aged between 6 and 23 months and 29 d who received breast milk on the day prior to the evaluation.	Children aged between 6 and 23 months and 29 d who received breast milk Total of children aged between 6 and 23 months and 29 d
* Introduction of solid, semi-solid or soft foods (ISSSF)	Percentage of children aged between 6 and 8 months and 29 d who consumed solid, semi-solid or soft foods on the previous day	Children aged between 6 and 8 months and 29 d who received solid, semi-solid or soft foods Total of children aged between 6 and 23 months and 29 d
Minimum meal frequency (MMF)**	– If a child aged 6–23 months and 29 d, consider the consumption of salty food at least once a day with a normal consistency (in pieces), or mashed. – If a child aged 7–23 months and 29 d, consider the consumption of salty food at least twice a day with a normal consistency (in pieces) or mashed.	Children aged between 6 and 23 months and 29 d who consumed salty food at the adequate frequency and consistency Total of children aged between 6 and 23 months and 29 d
Minimum dietary diversity (MDD)**	Percentage of children aged 6–23 months and 29 d who consumed the six related food groups on the day prior to the evaluation: 1. Breast milk or other non-breast milk, porridge with milk or yoghurt. 2. Fruits, legumes and vegetables. 3. Orange-coloured fruit or vegetables and dark green leaves. 4. Meat and eggs. 5. Beans; 6. Cereals and tubers (rice, potatoes, yam, cassava, flour or pasta – not instant).	Children aged between 6 and 23 months and 29 d who received six food groups Total of children aged between 6 and 23 months and 29 d
Minimum acceptable diet (MAD)**	Percentage of children aged 6–23 months and 29 d: – For breastfeed children: received at least minimum diet diversity and minimum meal frequency for their age during the previous day; – For children who are not breastfeed: received at least the minimum diet diversity and minimum meal frequency for their age during the previous day and at least two meals of milk.	Children aged 6–23 months and 29 d who consumed a minimally acceptable diet Total of children aged between 6 and 23 months and 29 d
* Consumption of meat and/or eggs	Percentage of children aged 6–23 months and 29 d who consumed meat and/or eggs.	Children aged between 6 and 23 months and 29 d who consumed meat and/or eggs Total of children aged between 6 and 23 months and 29 d
* Consumption of sweetened beverages	Percentage of children aged 6–23 months and 29 d who answered “Yes” to the question “Yesterday the child consumed sweetened drinks (soft drinks, carton juice, powdered juice, carton coconut water, guarana/currant syrups, and fruit juice with added sugar)?”	Children aged between 6 and 23 months and 29 d who consumed sweetened drinks Total of children aged between 6 and 23 months and 29 d
* Consumption of ultra-processed foods (UPF)	Percentage of children aged between 6 and 23 months and 29 d who consumed at least one of the following foods: – Hamburger and/or processed meat (ham, mortadella, salami, sausages and hot dogs). – Instant noodles, packaged snacks or crackers; – Sandwich biscuits, sweets or candies (sweets, lollipops, chewing gum, caramel and jelly).	Children aged between 6 and 23 months and 29 d who consumed ultra-processed foods Total of children aged between 6 and 23 months and 29 d
Consumption of Fe-rich foods**	Children aged 6–23 months and 29 d who consumed the three types of related foods: – Eggs or meat (beef, chicken, fish, pork, offal and others); – Liver; – Beans.	Children aged between 6 and 23 months and 29 d who received Fe-rich foods Total of children aged between 6 and 23 months and 29 d
Consumption of foods rich in vitamin A**	Consider all of the children in the age range with the answer “Yes” to the question “Yesterday the child ate a fruit or vegetable which was orange in colour (pumpkin, carrot, papaya and mango), or with dark green leaves (kale, caruru (okra stew), purslane, malabar spinach, spinach, and mustard greens)?”	Children aged between 6 and 23 months and 29 d who received foods rich in vitamin A Total of children aged between 6 and 23 months and 29 d
Zero consumption of fruit and vegetables**	Percentage of children aged between 6 and 23 months who did not consume any fruit or vegetables on the previous day,	Children aged between 6 and 23 months and 29 d who did not consume any fruit or vegetables Total of children aged between 6 and 23 months and 29 d
*Used for the time period 2008–2019. **Used for the time period 2015–2019.		

### Demographic and socio-economic variables

The socio-demographic data were obtained from SISVAN to characterise the study population: sex (female/male); age range (0–5 months/6–23 months) and geographic region of residence (North/Northeast/Central-West/Southeast/South). The BDI was used to evaluate the influence of the socio-economic profile of the child’s municipality of residence on indicators of feeding practices. The BDI is a measure that measures levels of material deprivation or, more generally, levels of socio-economic positions in different geographic areas of Brazil. This index was calculated based on three variables – (1) percentage of homes with a per capita income of ≤ 1/2 minimum salary; (2) percentage of illiterate people over the age of 7 and (3) average percentage of people with difficult access to sewage, water, waste collection, and do not have a bath/shower – in a single measure^(28)^. The measure was organised in descending order from the first quintile (less deprivation) to the last quintile (greater deprivation). The measure was validated by comparing it to other similar indices measuring health and social vulnerability at the census sector level in states and municipalities, and at the municipal level for across the whole of Brazil^(29)^. At the municipal level, the deprivation measure was also compared to health outcomes. The different validation exercises showed that the developed measure produced expected results and could be considered validated.

### Statistical analysis

The descriptive analysis of the study population’s characteristics was conducted through absolute and relative frequencies. The prevalence of indicators of feeding practices was estimated annually by demographic and socio-economic variables. Area graphics were used to illustrate how breastfeeding practices progressed as children grow. This graph is useful to understand exclusive breastfeeding patterns in different age ranges in the 0- to 5-month window and provides information on the types of other food introduced in this period in addition to breast milk for each age.

Prais–Winsten estimation was used to analyse the time trends in the prevalence of indicators of feeding practices. This generalised linear regression method has been widely used to correct serial correlations in time series^(30)^. The annual prevalence for each indicator was converted into a logarithmic scale to reduce heterogeneity of variance in the regression model. The prevalence values transformed into a logarithmic scale were defined as dependent variables, while the year was defined as the independent variable. The annual percentage variation (APC), and respective 95 % CI, were calculated in accordance with the following formula: APC = (–1 + 10*β*) × 100, where *β* is the Prais–Winsten regression coefficient^(30)^.

The ratio between the estimates of the extreme groups (quintiles) of the deprivation index was calculated. This is a simpler measure of relative inequality. This was calculated by dividing the prevalence values corresponding to the group with less deprivation (BDI/Q1) by the group with more deprivation (BDI/Q5). It produces the surplus percentage of one category in relation to the other, or how many times prevalent one group is compared to the other. All data were processed and analysed using Stata version 15·1 software (StataCorp. 2017. Stata Statistical Software: Release 15. StataCorp LLC).

### Complementary analysis

Time trend analyses of the prevalence of breastfeeding and complementary feeding practice were conducted by quintiles of the Municipal Human Development Index (MHDI). Education, longevity and income are considered in this index^(31)^, and it varies from 0 to 1. The closer to 1, the higher the human development.

## Results

The study included a total of 911 735 children, with a variation of 12 279 in 2008 and 115 063 in 2019. We found a slight increase in the coverage of food intake monitoring over the years, ranging from 0·21 to 2·33 %. Half of the children evaluated were male (50·3 %), and the majority were aged between 6 and 23 months. 8·7 % were from the North, 18·8 % from the Northeast, 14·1 % from the South, 11·6 % from the Central-West and 46·5 % the Southeast region. Regarding the level of deprivation, 29·6 % of the children evaluated were in the quintile of highest municipal deprivation (Q5). Further characteristics of the study population can be found in Table 1.

**Table 1:** Characteristics of the study population. Food and Nutrition Surveillance System (SISVAN), Brazil, 2008–2019

	Time series													
Characteristics		2008	2009	2010	2011	2012	2013	2014	2015	2016	2017	2018	2019	Total
Total		12 279	28 449	29 736	43 026	61 304	64 593	81 285	66 391	138 647	134 607	136 355	115 063	911 735
Coverage		0·21	0·48	0·51	0·74	1·05	1·11	1·11	1·39	1·94	2·29	2·31	2·33	
Sex														
Female	*n*	6153	14 334	14 712	21 668	31 155	30 968	43 186	32 827	68 824	66 433	66 571	56 375	453 206
	%	50·1	50·4	49·5	50·4	50·8	47·9	53·1	49·4	49·6	49·3	48·8	49·0	49·7
Male	*n*	6126	14 115	15 024	21 358	30 149	33 625	38 099	33 564	69 823	68 174	69 784	58 688	458 529
	%	49·9	49·62	50·5	49·6	49·2	52·1	46·9	51·6	50·4	50·7	51·2	51·0	50·3
Age														
0–5 months	*n*	4041	11 204	12 595	20 513	30 095	30 926	42 556	29 553	58 059	62 742	63 496	50 720	416 500
	%	32·9	39·4	42·4	47·7	49·1	47·9	52·3	44·5	41·9	46·6	46·6	44·1	45·7
6–23 months	*n*	8238	17 245	17 141	22 513	31 209	33 667	38 729	36 838	80 588	71 865	72 859	64 343	495 235
	%	67·1	60·6	57·6	52·3	50·9	52·1	47·7	55·5	58·1	53·4	53·4	55·9	54·3
Region of residence														
North	*n*	1717	2632	2755	2866	5497	5651	8820	7587	11 162	6279	8074	7843	70 883
	%	14·0	9·2	9·26	6·66	8·9	8·7	10·8	11·4	8·1	4·7	5·9	6·8	7·8
Northeast	*n*	2166	4231	6234	7222	12 249	10 500	10 211	12 876	28 939	16 650	30 421	39 244	180 943
	%	17·6	14·9	20·9	16·8	19·9	16·3	12·6	19·4	20·9	12·4	22·3	34·1	19·8
Southeast	*n*	4863	10 574	12 478	14 026	25 929	32 318	39 844	27 170	73 069	92 023	75 962	54 702	462 958
	%	39·6	37·2	41·9	32·6	42·3	50·0	49·0	40·9	52·7	68·4	55·7	47·6	50·8
South	*n*	2567	5114	3390	8276	8223	8706	11 900	10 897	14 353	13 994	16 916	10 610	114 946
	%	20·9	17·9	11·4	19·2	13·4	13·5	14·6	16·4	10·4	10·4	12·4	9·2	12·6
Central-West	*n*	966	5898	4879	10 636	9406	7418	10 510	7861	11 124	5661	4982	2664	82 005
	%	7·9	20·7	16·5	24·7	15·3	11·5	12·9	11·8	8·0	4·2	3·7	2·3	9·0
Brazilian deprivation index														
Q1	*n*	3619	4493	6244	7208	11 195	8084	11 045	13 509	18 625	19 242	16 448	10 310	130 022
	%	29·5	15·8	21·0	16·8	18·3	12·5	13·6	20·4	13·4	14·3	12·1	9·0	14·3
Q2	*n*	746	4456	4033	8299	12 231	12 833	20 902	9279	13 390	15 917	14 877	10 321	127 284
	%	6·1	15·7	13·6	19·3	20·0	19·9	25·7	14·0	9·7	11·8	10·9	9·0	14·0
Q3	*n*	1571	6432	5972	11 240	11 610	12 866	14 686	13 267	33 349	35 502	30 898	20 526	197 919
	%	12·8	22·6	20·1	26·1	18·8	19·9	18·1	20·0	24·1	26·4	22·7	17·8	21·7
Q4	*n*	2844	6214	6200	7206	9539	12 177	17 003	10 188	31 144	30 312	29 431	24 258	186 516
	%	23·2	21·8	20·9	16·8	15·6	18·9	20·9	15·4	22·5	22·5	21·6	21·1	20·5
Q5	*n*	3499	6854	7287	9073	16 729	18 633	17 649	20 148	42 139	33 634	44 701	49 648	269 994
	%	28·5	24·1	24·5	21·1	27·3	28·9	21·7	30·4	30·4	25·0	32·8	43·2	29·6

**Fig. 2:**
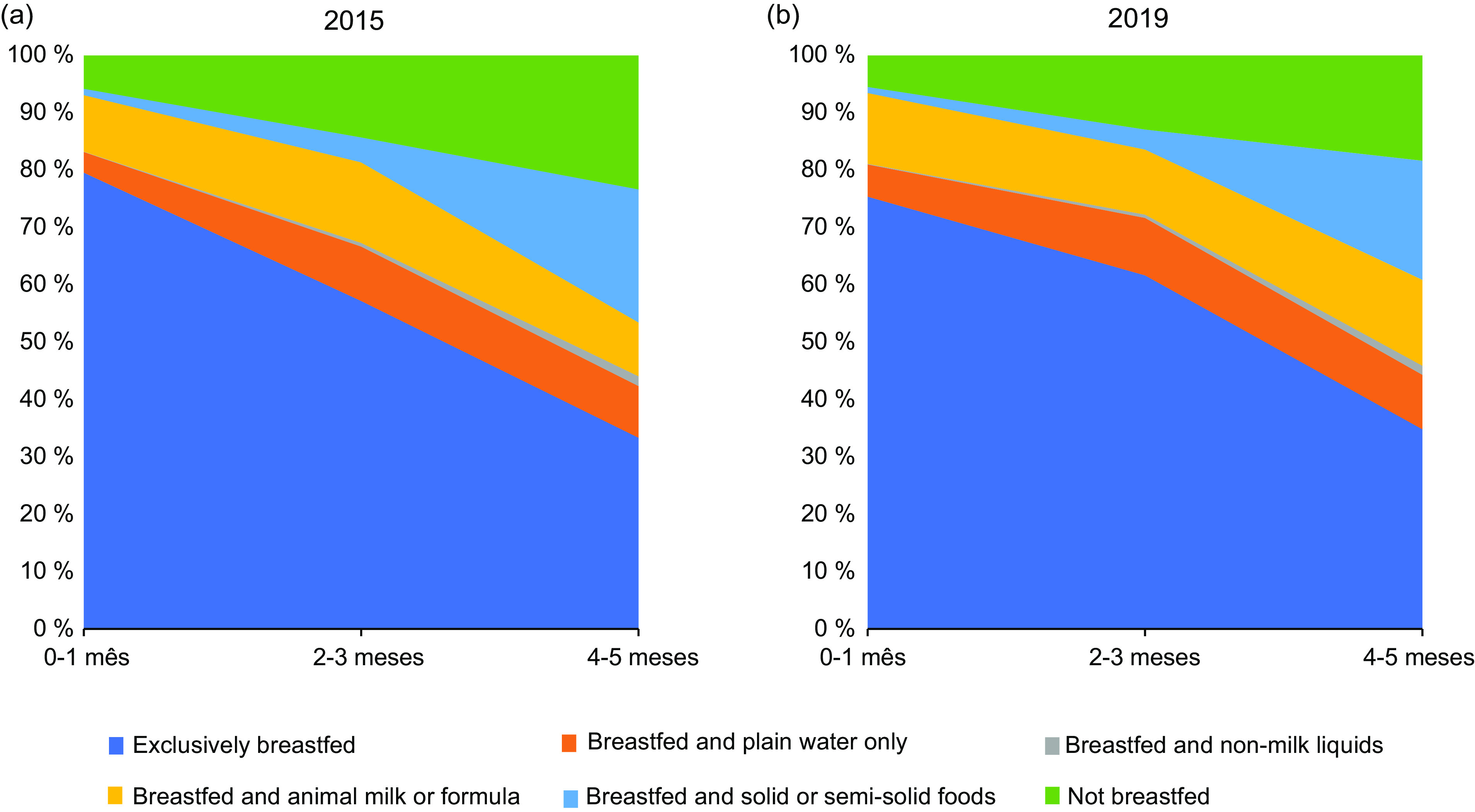
Infant feeding area graphs under 6 months - Food and Nutritional Surveillance System in 2015 and 2019

The prevalence of breastfeeding practices in children under 6 months of age in 2015 and in 2019 are presented in Fig. 2. In the first month of life, more than 70 % of children were exclusively breastfed. This prevalence reduced progressively until 4 and 5 months in both years. From 2 to 3 months, there was an expressive increase in the prevalence of ISSSF. In addition, a considerable percentage of the children consumed breast milk and water. Mixed breastfeeding also increased during the period analysed.

Time-trend analysis of the prevalence of feeding practice indicators, in accordance with the BDI, can be found in Table 2 and Table 3. Overall, the percentage of children under 6 months in mixed breastfeeding varied from 10·0 % to 11·9 % between 2015 and 2019, corresponding to an annual variation of +10·38 % (*P* = 0·013). This increase was demonstrated in the extreme BDI quintiles, especially in the municipalities with more deprivation (Q1: APC = +10·60, *P* = 0·003; and Q5: APC = +21·74, *P* = 0·002).

**Table 2:** Prevalence of breast-feeding practice indicators in children under 24 months in accordance with the Brazilian deprivation index. Food and Nutrition Surveillance System (SISVAN), Brazil, 2008–2019

Indicators		Time series												APC	95 % CI	*P*-value	R^2^
		2008	2009	2010	2011	2012	2013	2014	2015	2016	2017	2018	2019				
Exclusive breast-feeding	Total	43·6	63·9	56·6	59·9	58·3	*	50·8	57·0	54·0	59·5	57·4	55·38	0·56	−2·79, 4·02	0·719	0·98
	q1	23·8	69·3	59·5	62·2	61·5	–	50·3	64·2	60·9	67·2	63·2	63·7	7·80	−2·46, 19·13	0·123	0·85
	q5	42·1	67·7	57·6	64·5	50·3	–	46·4	49·4	49·9	55·0	50·8	50·7	−2·52	−7·24, 2·43	0·274	0·95
Ratio Q1/Q5		0·57	1·02	1·03	0·96	1·22	–	1·08	1·30	1·22	1·22	1·24	1·26				
Mixed breast-feeding	Total	–	–	–	–	–	–	–	10·0	10·7	11·5	11·6	11·9	+10·38	4·05, 17·10	0·013	0·70
	q1	–	–	–	–	–	–	–	12·1	13·5	13·2	14·2	15·0	+10·60	6·63, 14·71	0·003	1·00
	q5	–	–	–	–	–	–	–	7·4	8·1	8·3	9·5	10·5	+21·74	14·48, 29·46	0·002	1·00
Ratio Q1/Q5		–	–	–	–	–	–	–	1·64	1·67	1·59	1·49	1·43				
Continued breast-feeding	Total	52·0	55·5	50·5	51·1	51·1	52·3	46·3	57·7	51·2	52·8	56·7	58·9	1·34	−1·07, 3·81	0·246	0·95
	q1	64·6	52·2	58·1	40·3	39·7	45·9	43·2	45·7	47·0	49·6	51·7	52·9	−2·43	−10·21, 6·02	0·524	0·92
	q5	53·1	62·1	52·6	60·8	58·8	57·6	56·2	57·0	56·4	57·4	60·8	61·7	0·92	−0·50, 2·36	0·179	1·00
Ratio Q1/Q5		1·22	0·84	1·10	0·66	0·68	0·80	0·77	0·80	0·83	0·86	0·85	0·86				

APC, annual percentage change; R^2^, coefficient of determination.

* Exclusive breastfeeding data could not be used, since information was missing for 2013.

**Table 3:** Prevalence of complementary feeding practice indicators in children under 24 months in accordance with the Brazilian deprivation index. Food and Nutrition Surveillance System (SISVAN), Brazil, 2008–2019

Indicators	Time series													APC	95 % CI	*P*-value	R^2^
		2008	2009	2010	2011	2012	2013	2014	2015	2016	2017	2018	2019				
Introduction of solid, semi-solid or soft foods (6 – 8 months)	Total	85·1	85·8	84·1	82·7	84·1	82·3	80·4	83·2	84·8	85·9	85·2	84·8	0·02	−1·20, 1·26	0·973	1·00
	q1	96·0	90·7	96·7	88·6	91·2	92·0	94·0	87·0	87·5	86·1	87·8	86·6	−1·90	−2·69, −1·11	< 0·001	1·00
	q5	76·2	75·9	73·7	70·1	74·4	69·8	55·7	78·7	86·4	83·6	82·1	82·5	2·65	−3·11, 8·77	0·337	0·87
Ratio Q1/Q5		1·26	1·19	1·31	1·26	1·23	1·32	1·69	1·11	1·01	1·03	1·07	1·05				
Minimum meal frequency	Total	–	–	–	–	–	–	–	70·0	72·3	74·2	72·1	70·1	0·04	−6·50, 7·03	0,987	0·98
	q1	–	–	–	–	–	–	–	79·6	80·1	79·5	79·6	79·9	−0·11	−0·65, 0·43	0,553	1·00
	q5	–	–	–	–	–	–	–	62·7	65·2	67·3	64·1	64·2	0·69	−5·82, 7·65	0,766	0·97
Ratio Q1/Q5		–	–	–	–	–	–	–	1·27	1·23	1·18	1·24	1·24				
Minimum dietary diversity (6–23 months)	Total	–	–	–	–	–	–	–	39·6	41·8	44·0	40·7	39·8	−0·43	−11·35, 11·82	0·913	0·02
	q1	–	–	–	–	–	–	–	47·8	48·1	48·9	47·6	52·2	+1·44	0·97, 1·91	0·006	0·99
	q5	–	–	–	–	–	–	–	30·1	32·3	35·5	32·2	31·5	2·09	−12·69, 19·39	0·702	0·20
Ratio Q1/Q5		–	–	–	–	–	–	–	1·59	1·49	1·38	1·47	1·66				
Minimum acceptable diet	Total	–	–	–	–	–	–	–	28·6	38·9	32·8	30·5	29·5	−6·82	−26·13, 17·54	0·404	0·99
	q1	–	–	–	–	–	–	–	34·5	34·9	36·1	35·8	40·5	+5·17	3·67, 6·70	0·004	0·99
	q5	–	–	–	–	–	–	–	21·2	23·7	26·1	23·4	22·8	3·15	−15·11, 25·35	0·647	.
Ratio Q1/Q5		–	–	–	–	–	–	–	1·63	1·47	1·38	1·53	1·78				
Consumption of meat and/or eggs	Total	63·9	66·3	66·8	71·5	70·2	70·88	76·6	76·2	76·6	76·9	76	74·9	+3·68	1·83, 5·57	0·001	0·99
	q1	59·7	63·0	67·9	74·6	67·1	79·4	77·7	80·6	81·0	79·9	80·4	80·3	+6·26	3·55, 9·04	< 0·001	0·61
	q5	65·7	67·1	63·8	61·8	64·7	63·5	68·2	70·5	70·4	71·8	70·8	70·7	+2·20	0·11, 4·33	0·041	1·00
Ratio Q1/Q5		0·91	0·94	1·06	1·21	1·04	1·25	1·14	1·14	1·15	1·11	1·14	1·14				
Consumption of sweetened drinks	Total	57·9	62·2	60·1	58·8	54·5	49·3	53·8	39·7	36·9	33·6	31·3	30·72	−14·74	−18·68, −10·61	< 0·001	0·95
	q1	56·1	64·7	56·9	66·8	54·4	61·7	55·9	41·4	36·1	33·3	33·9	30·5	−14·39	−20·65, −7·64	0·001	0·91
	q5	55·8	61·9	53·6	52·6	50·8	46·6	47·2	37·8	36·1	32·5	30·3	28·8	−14·84	−17·26, −12·36	< 0·001	0·94
Ratio Q1/Q5		1·01	1·05	1·06	1·27	1·07	1·32	1·18	1·10	1·00	1·02	1·12	1·06				
Consumption of ultra-processed foods	Total								41·9	40·1	37·6	35·9	35·5	−9·96	−12·75, −7·09	0·002	1·00
	q1	–	–	–	–	–	–	–	42·4	40·6	37·9	38·9	35·7	−7·76	−10·71, −4·72	0·004	1·00
	q5	–	–	–	–	–	–	–	41·7	40·5	38·0	36·3	34·8	−10·56	−11·52, −9·58	< 0·001	1·00
Ratio Q1/Q5		–	–	–	–	–	–	–	1·02	1·00	1·00	1·07	1·03				
Consumption of Fe-rich foods	Total								12·9	12·9	12·3	11·9	12·1	−5·67	−8·13, −3·14	0·006	1·00
	q1	–	–	–	–	–	–	–	12·1	15·1	14·2	13·7	14·4	3·88	−10·42, 20·48	0·473	0·99
	q5	–	–	–	–	–	–	–	12·3	11·3	11·2	11·17	11·8	−2·10	−10·87, 7·53	0·523	0·97
Ratio Q1/Q5		–	–	–	–	–	–	–	0·98	1·34	1·27	1·23	1·22				
Consumption of foods rich in vitamin A	Total	–	–	–	–	–	–	–	65·8	67·4	67·9	64·9	63·7	−2·34	−7·63, 3·26	0·270	0·38
	q1	–	–	–	–	–	–	–	74·7	73·1	71·5	70·9	73·8	−1·43	−5·98, 3·35	0·405	1·00
	q5	–	–	–	–	–	–	–	57·3	59·8	62·1	58·5	57·1	−0·65	−9·46, 9·03	0·838	0·52
Ratio Q1/Q5		–	–	–	–	–	–	–	1·30	1·22	1·15	1·21	1·29				
Zero consumption of fruit and vegetables (6–23 months)	Total	14·3	12·4	10·5	12·4	10·1	11·1	8·7	8·9	8·2	7·2	8·24	9·4	−10·53	−14·33, −6·56	< 0·001	0·68
	q1	3·6	5·8	3·6	6·3	3·7	4·9	9·2	4·8	5·0	5·1	5·4	5·6	5·05	−2·51, 13·20	0·172	0·72
	q5	19·9	19·0	17·6	21·3	16·2	17·2	14·7	13·9	12·9	10·7	11·9	12·6	−12·15	−15·35, −8·83	< 0·001	0·84
Ratio Q1/Q5		0·18	0·30	0·21	0·29	0·23	0·29	0·63	0·35	0·39	0·47	0·45	0·45				

APC, annual percentage change; R^2^, coefficient of determination.

With regards to complementary feeding indicators, a low prevalence of MDD and MAD indicators was observed in all years of the series studied. It was also noted that the percentage of children in compliance with the ISSSF indicator reduced in the municipalities with less deprivation (Q1: –1·90, *P* < 0·001). These results were reversed for MDD (Q1: +1·44, *P* = 0·006) and MAD (Q1: +5·17, *P* = 0·004).

A decrease in the percentage of children who consume UPF (Q1: –7·76, *P* = 0·004; and Q5: –10·56, *P* < 0·001) and sweetened beverages (Q1: –14·39, *P* = 0·001; and Q5: –14·84, *P* < 0·001) was registered, independently of the extreme BDI quintiles. An increase in the percentage of children who consume meat and/or eggs was observed, also independently of the extreme BDI quintiles, although higher in the municipalities with less deprivation (Q1: +6·29, *P* < 0·001; and Q5: +2·20, *P* = 0·041). In general, the percentage of children who consume Fe-rich foods reduced over time (–5·67, *P* = 0·006). The percentage of children who do not consume fruit and vegetables also reduced (–10·53, *P* < 0·001), especially in the municipalities with more deprivation (Q5: –12·15, *P* < 0·001).

Similar trend patterns in the indicators of child feeding practices were found when evaluated by the MDHI (online Supplementary Table 1 and Table 2).

## Discussion

This study enabled the analysis of time trends in indicators of child feeding practices in Brazil, according to an important marker of social inequality. Our data indicated an increase in the proportion of children under 6 months experiencing mixed breastfeeding, especially in the municipalities with more poverty, and a low prevalence of MDD and MAD indicators. Furthermore, breastfeeding patterns and complementary feeding differed among extreme BDI quintiles. In general, results were more favourable in municipalities with less poverty. Improvements in complementary feeding indicators, such as MDD, MAD and the consumption of meat and/or eggs, were primarily demonstrated in the municipalities with less poverty. The reduction in the proportion of children who did not consume fruit and vegetables were more accentuated in the municipalities with more poverty. The decrease in the consumption of sweetened beverages and UPF, independently of the degree of inequality, was also highlighted.

Despite the recommendation that breast milk should be the only food offered to children under 6 months, we found that almost 30 % of children abandon exclusive breastfeeding in the first month, an alert for the intensification of actions to promote exclusive breastfeeding in primary care in the first weeks of life. Our data did not indicate any trend in the prevalence of exclusive breastfeeding, which remained stable in the 50–60 % range for the entire series, although higher than those found by ENANI, 2019 (45·8 %)^(12)^. The differences in these results can be explained by the type of population to which our study refers. The downturn in the gains which had been observed between 1986 and 2006 is cause for concern. For the first time in a historical series, real increases in the prevalence of exclusive breastfeeding in Brazil were not observed^(32)^. On the other hand, the increase in the proportion of children experiencing mixed breastfeeding, mainly in the municipalities with a higher level of deprivation, is cause for alarm. This practice has been associated with poor oral health and increased risk of overweight/obesity^(33)^.

An increasing trend in MAD prevalence in the municipalities with less poverty was confirmed. As an indicator which includes breast milk substitutes, MAD implies that children need to receive both diverse foods (MDD) in a recommended number of meals (minimum meal frequency) such as the consumption of six food groups a day. This condition may not be easy to achieve in poorer regions^(34)^. Certainly, many families still face the challenge of meeting minimum dietary standards for children^(35)^. This highlights the need for a broader understanding of complementary feeding practices in the context of food and nutrition insecurity^(36,37)^. Similar negative associations between MAD and low socio-economic status were also found in others studies, which used Demographic and Health Surveys data, conducted in low- and middle-income countries^(19,34,38)^.

We observed a reduction in the percentage of children aged 6–24 months who consumed sweetened beverages and UPF, independently of the extreme poverty quintiles. In parallel, an important reduction in the proportion of children who do not consume fruit or vegetables was noted, especially in municipalities with more poverty. Shifts in feeding practices have been observed in recent decades, inserted in the concept of nutrition transition, which is a process of sequential changes in dietary patterns. Therefore, a dietary pattern does not remain stagnant. We can speculate about the emergence of a new stage of the nutrition transition process, as a consequence of the desire to prevent or delay degenerative diseases and extend good health, with the adoption of a better quality diet and higher amount of fruit, vegetables and whole grains^(39)^.

Despite the important reduction in the consumption of UPF and sweetened drinks, their prevalence remain very high in this population, corroborating the findings of previous national and international studies^(40–42)^. The consumption of UPF in the first 2 years of life is not recommended, since they usually have a high energy density, higher quantity of sugar, Na, saturated fat and lower quantity of essential fibres and nutrients^(43,44)^. However, the high palatability, availability and ‘aggressive’ marketing of these products challenge conscientious consumption and make them preferential replacements for in natura, or minimally processed foods^(43,45)^. Another aggravating factor is that the introduction of these products has been taking place very prematurely in children’s diets, even before they reach 12 months of age. The consumption of UPF is also related to a higher prevalence of obesity, chronic diseases and nutritional deficiencies in the early years of life and may also compromise the consumption of the healthy foods associated with adequate growth and child development^(46––48)^. On account of this, special attention must be given to the consumption of complementary foods in this life stage, in accordance with the Food Guide for the Brazilian population^(49)^, an official document released by the country’s Ministry of Health. The guide provides recommendations and information on feeding practices in the first 2 years of life, with the aim of promoting good health, growth and development, so that they are able to achieve their full potential.

An increase in the proportion of children who consume meat and/or eggs was observed, especially in the municipalities with less poverty. These disparities are probably due to the high cost of these foods^(50)^. Inaccessibility and the high cost of protein-rich foods have been a growing area of concern, preventing the adoption of adequate and healthy diets^(51)^. A global analysis involving 177 countries showed that a diet which is adequate in protein costs 2·66 times the cost of daily energy subsistence, and this value was much higher in Sub-Saharan Africa^(50)^. In Brazil, the price of protein-rich foods has increased significantly in recent years while the price of UPF has remained stable or even declined over the same period^(52)^. Therefore, these price changes may further discourage the adoption of a more diverse diet, which partly explains the prevalence of UPF remaining high for all social strata.

At the national level, health and nutrition interventions executed through large scale and multisectoral programmes are essential to address the inequalities shown in our results. Among these initiatives, we highlight the national breastfeeding and complementary feeding strategy (Amamenta e Alimenta Brasil), the Dietary Guidelines for Brazilian Children Under 2 Years of Age, the federal conditional cash transfer programme’s health conditions (monitoring of nutritional status, vaccination and prenatal visits) and the qualification of maternal, prenatal and child health care^(23,49,53)^. In addition, it may be useful to learn from successful actions implemented by other countries that have adopted an integrated strategy to promote breastfeeding and healthy complementary feeding by focusing on interventions during pregnancy and in the first 2 years of life.

### Strengths and limitations

As in all studies that use secondary data, limitations related to incompleteness, underestimation and classification bias should be recognised. Another limitation of this study resides in the fact that SISVAN data is not representative of the total population of Brazil. Primary Care covers around 60–70 % of the population and SISVAN covers less than 3 % of the population of children in the analysed age group. On the other hand, SISVAN constitutes a good data source for food and nutrition surveillance, which has advanced over time in coverage and data quality, representing an important tool for public policy management and the production of evidence in the field of maternal and child health. Another limitation of this study is the differences in the data collection forms in the years the research was conducted. Despite this point, the study provides relevant evidence on breastfeeding and the complementary feeding of Brazilian children over the years studied.

However, this study has the merit of identifying changes in the indicators of food consumption for children under 2 years of age over a 12-year period, using national data produced by an information system used by health care services and managed by the Brazilian Ministry of Health. We have used the indicators suggested by the WHO, and partly adopted by the Ministry of Health, which allows comparability with other national and international studies. Although the data collection instrument does not allow us to detail the frequency and age at which food was introduced, the questionnaire has important advantages such as the easy and fast application by any primary health care professional. The information collection technique also allows for the reduction of memory bias and is used by health teams to monitor the food consumption indicators for the population within the SUS. To the best of our knowledge, this is the first study to evaluate food consumption indicators in accordance with the BDI.

## Conclusion

Breastfeeding and complementary feeding patterns differ among the extreme BDI quintiles. In general, results were more favourable in municipalities with less poverty. Data also demonstrated improvements to these patterns over time, especially in municipalities with less poverty. Inadequate complementary feeding practices are the main determinants of malnutrition, development and mortality. Therefore, these findings have the potential to assist in the preparation of targeted actions for high-risk groups. They also contribute towards prevention and control of morbidities associated with diet, thereby supporting attaining the Sustainable Development Goals described in the United Nations 2030 agenda, including efforts to eradicate hunger and malnutrition, and to promote health and well-being.

## Supplementary material

For supplementary material accompanying this paper visit https://doi.org/10.1017/S1368980023001039. click here to view supplementary material
