# Supplementary material for: Time trends and social inequalities in infant and young child feeding practices: national estimates from Brazil’s Food and Nutrition Surveillance System, 2008–2019
Source: Public Health Nutr. 2023 May 26;26(9):1731–42. doi: 10.1017/S1368980023001039 (PMC10478053; doi:10.1017/S1368980023001039)
Supplement: Supplementary file 1 [file S1368980023001039sup001.docx]

**Supplementary Table 1.** Prevalence of breastfeeding practice indicators in children under 24 months in accordance with the Municipal Human Development Index. Food and Nutrition Surveillance System (SISVAN), Brazil, 2008–2019.

| **Indicators** |  | **Time series** | | | | | | | | | | | | **APC** | **95% CI** | | **p-value** | **R^2^** |
| --- | --- | --- | --- | --- | --- | --- | --- | --- | --- | --- | --- | --- | --- | --- | --- | --- | --- | --- |
|  |  | **2008** | **2009** | **2010** | **2011** | **2012** | **2013** | **2014** | **2015** | **2016** | **2017** | **2018** | **2019** |  |  |  |  |  |
| **Exclusive breastfeeding** | Total | 43.6 | 63.9 | 56.6 | 59.9 | 58.3 | * | 50.8 | 57.0 | 54.0 | 59.5 | 57.4 | 55.38 | 0.56 | -2.79 | 4.02 | 0.719 | 0.98 |
|  | q5 | 24.7 | 69.3 | 58.0 | 63.3 | 64.6 | - | 48.9 | 63.3 | 60.6 | 64.5 | 62.2 | 24.7 | 6.77 | -3.25 | 17.84 | 0.167 | 0.85 |
|  | q1 | 47.1 | 70.5 | 58.3 | 66.3 | 49.7 | - | 42.9 | 48.3 | 47.7 | 53.5 | 47.6 | 47.1 | -5.11 | -9.72 | -0.28 | 0.041 | 0.95 |
| Ratio Q5/Q1 |  | 0.52 | 0.98 | 1.00 | 0.96 | 1.30 |  | 1.14 | 1.31 | 1.27 | 1.20 | 1.31 | 0.52 |  |  |  |  |  |
| **Mixed breastfeeding** | Total | - | - | - | - | - | - | - | 10.0 | 10.7 | 11.5 | 11.6 | 11.9 | 10.38 | +4.05 | 17.10 | **0.013** | 0.70 |
|  | q5 | - | - | - | - | - | - | - | 11.1 | 12.5 | 12.9 | 12.2 | 11.1 | 8.26 | -0.66 | 17.97 | 0.061 | 1.00 |
|  | q1 | - | - | - | - | - | - | - | 6.8 | 7.4 | 7.2 | 8.6 | 6.8 | 24.48 | +6.65 | 45.29 | **0.020** | 0.91 |
| Ratio Q5/Q1 |  | - | - | - | - | - | - | - | 1.6 | 1.7 | 1.8 | 1.4 | 1.6 |  |  |  |  |  |
| **Continued breastfeeding** | Total | 52.0 | 55.5 | 50.5 | 51.1 | 51.1 | 52.3 | 46.3 | 57.7 | 51.2 | 52.8 | 56.7 | 58.9 | 1.34 | -1.07 | 3.81 | 0.246 | 0.95 |
|  | q5 | 63.2 | 57.2 | 45.8 | 51.3 | 44.4 | 50.7 | 52.8 | 50.7 | 49.9 | 52.1 | 53.3 | 55.96 | -0.89 | -5.65 | 4.12 | 0.696 | 0.91 |
|  | q1 | 57.5 | 60.5 | 54.3 | 62.6 | 60.2 | 57.4 | 56.1 | 58.3 | 58.2 | 58.7 | 61.6 | 62.11 | 0.80 | -0.76 | 2.37 | 0.283 | 0.98 |
| Ratio Q5/Q1 |  | 1.10 | 0.95 | 0.84 | 0.82 | 0.74 | 0.88 | 0.94 | 0.87 | 0.86 | 0.89 | 0.86 | 0.90 |  |  |  |  |  |

APC: annual percentage change. 95% CI: 95% confidence interval. R^2^: coefficient of determination.

* Exclusive breastfeeding data could not be used, since information was missing for 2013.

**Supplementary Table 2.** Prevalence of complementary feeding practice indicators in children under 24 months in accordance with the Municipal Human Development Index. Food and Nutrition Surveillance System (SISVAN), Brazil, 2008–2019.

| **Indicators** | **Time series** | | | | | | | | | | | | | **APC** | **95% CI** | | **p-value** | **R^2^** |
| --- | --- | --- | --- | --- | --- | --- | --- | --- | --- | --- | --- | --- | --- | --- | --- | --- | --- | --- |
|  |  | **2008** | **2009** | **2010** | **2011** | **2012** | **2013** | **2014** | **2015** | **2016** | **2017** | **2018** | **2019** |  |  |  |  |  |
| Introduction of solid, semi-solid, or soft foods (6 – 8 months) | Total | 85.1 | 85.8 | 84.1 | 82.7 | 84..1 | 82.3 | 80.4 | 83.2 | 84.8 | 85.9 | 85.2 | 84.8 | 0.02 | -1.20 | 1.26 | 0.973 | 1.00 |
|  | q5 | 95.7 | 82.1 | 87.8 | 81.9 | 87.2 | 84.7 | 87.7 | 83.4 | 85.7 | 85.4 | 87.7 | 87.2 | -0.13 | -1.25 | 1.00 | 0.807 | 1.00 |
|  | q1 | 77.2 | 71.0 | 75.7 | 66.1 | 68.9 | 72.0 | 57.8 | 78.1 | 80.0 | 82.0 | 81.2 | 81.2 | 2.74 | -1.79 | 7.48 | 0.211 | 0.54 |
| **Ratio Q5/Q1** |  | 1.24 | 1.16 | 1.16 | 1.24 | 1.27 | 1.18 | 1.52 | 1.07 | 1.07 | 1.04 | 1.08 | 1.07 |  |  |  |  |  |
| Minimum meal frequency | Total | - | - | - | - | - | - | - | 70.0 | 72.3 | 74.2 | 72.1 | 70.1 | 0.04 | -6.50 | 7.03 | 0.987 | 0.98 |
|  | q5 | - | - | - | - | - | - | - | 73.9 | 78.0 | 76.1 | 77.4 | 77.1 | 1.32 | -1.01 | 3.70 | 0.170 | 1.00 |
|  | q1 | - | - | - | - | - | - | - | 60.2 | 60.6 | 64.9 | 62.4 | 62.7 | 2.66 | -2.84 | 8.47 | 0.226 | 1.00 |
| **Ratio Q5/Q1** |  | - | - | - | - | - | - | - | 1.23 | 1.29 | 1.17 | 1.24 | 1.23 |  |  |  |  |  |
| Minimum dietary diversity (6 – 23 months) | Total | - | - | - | - | - | - | - | 39.6 | 41.8 | 44.0 | 40.7 | 39.8 | -0.43 | -11.35 | 11.82 | 0.913 | 0.02 |
|  | q5 | - | - | - | - | - | - | - | 46.4 | 47.7 | 48.9 | 47.2 | 48.8 | 1.67 | -1.73 | 5.20 | 0.219 | 1.00 |
|  | q1 | - | - | - | - | - | - | - | 26.8 | 24.4 | 31.9 | 28.6 | 27.8 | 7.63 | -8.98 | 27.27 | 0.257 | 1.00 |
| **Ratio Q5/Q1** |  | - | - | - | - | - | - | - | 1.73 | 1.95 | 1.53 | 1.65 | 1.76 |  |  |  |  |  |
| Minimum acceptable diet | Total | - | - | - | - | - | - | - | 28.6 | 38.9 | 32.8 | 30.5 | 29.5 | -6.82 | -26.13 | 17.54 | 0.404 | 0.99 |
|  | q5 | - | - | - | - | - | - | - | 33.7 | 35.7 | 36.2 | 35.9 | 37.4 | +4.56 | 0.94 | 8.31 | **0.027** | 1.00 |
|  | q1 | - | - | - | - | - | - | - | 18.2 | 19.3 | 22.9 | 20.9 | 19.7 | 5.68 | -14.11 | 30.03 | 0.459 | 0.61 |
| **Ratio Q5/Q1** |  | - | - | - | - | - | - | - | 1.85 | 1.85 | 1.58 | 1.72 | 1.90 |  |  |  |  |  |
| Consumption of meat and/or eggs | Total | 63.9 | 66.3 | 66.8 | 71.5 | 70.2 | 70.88 | 76.6 | 76.2 | 76.6 | 76.9 | 76.0 | 74.9 | +3.68 | 1.83 | 5.57 | **0.001** | 0.99 |
|  | q5 | 60.8 | 68.7 | 75.9 | 73.7 | 71.6 | 76.1 | 81.4 | 78.9 | 80.0 | 78.3 | 78.9 | 78.8 | +4.45 | 1.48 | 7.50 | **0.007** | 0.97 |
|  | q1 | 60.6 | 67.5 | 64.4 | 59.8 | 59.2 | 59.8 | 67.7 | 68.5 | 68.1 | 70.6 | 68.8 | 68.7 | 2.75 | -0.22 | 5.82 | 0.066 | 0.98 |
| **Ratio Q5/Q1** |  | 1.00 | 1.02 | 1.18 | 1.23 | 1.21 | 1.27 | 1.20 | 1.15 | 1.17 | 1.11 | 1.15 | 1.15 |  |  |  |  |  |
| Consumption of sweetened drinks | Total | 57.9 | 62.2 | 60.1 | 58.8 | 54.5 | 49.3 | 53.8 | 39.7 | 36.9 | 33.6 | 31.3 | 30.72 | -14.74 | -18.68 | -10.61 | **<0.001** | 0.95 |
|  | q5 | 57.0 | 59.2 | 63.0 | 57.0 | 51.5 | 47.8 | 41.7 | 38.9 | 35.8 | 33.6 | 32.8 | 31.4 | -13.30 | -16.91 | -9.52 | **<0.001** | 0.99 |
|  | q1 | 58.6 | 59.2 | 56.4 | 51.5 | 55.1 | 44.8 | 45.6 | 37.1 | 36.7 | 32.1 | 29.9 | 29.14 | -15.39 | -17.53 | -13.20 | **<0.001** | 0.95 |
| **Ratio Q5/Q1** |  | 0.97 | 1.00 | 1.12 | 1.11 | 0.93 | 1.07 | 0.92 | 1.05 | 0.97 | 1.05 | 1.10 | 1.08 |  |  |  |  |  |
| Consumption of ultra- processed foods | Total |  |  |  |  |  |  |  | 41.9 | 40.1 | 37.6 | 35.9 | 35.5 | -9.96 | -12.75 | -7.09 | **0.002** | 1.00 |
|  | q5 | - | - | - | - | - | - | - | 39.5 | 38.8 | 36.4 | 36.7 | 35.7 | -5.88 | -8.66 | -3.00 | **0.008** | 1.00 |
|  | q1 | - | - | - | - | - | - | - | 41.6 | 40.5 | 38.9 | 36.7 | 35.32 | -9.40 | -11.15 | -7.62 | **0.001** | 1.00 |
| **Ratio Q5/Q1** |  | - | - | - | - | - | - | - | 0.95 | 0.96 | 0.94 | 1.00 | 1.01 |  |  |  |  |  |
| Consumption of iron-rich foods | Total |  |  |  |  |  |  |  | 12.9 | 12.9 | 12.3 | 11.9 | 12.1 | -5.67 | -8.13 | -3.14 | **0.006** | 1.00 |
|  | q5 | - | - | - | - | - | - | - | 12.6 | 14.7 | 12.8 | 12.5 | 12.71 | -5.50 | -14.30 | 4.21 | 0.163 | 1.00 |
|  | q1 | - | - | - | - | - | - | - | 10.7 | 10.7 | 11.3 | 11.2 | 10.98 | 2.46 | -2.18 | 7.33 | 0.194 | 0.99 |
| **Ratio Q5/Q1** |  | - | - | - | - | - | - | - | 1.18 | 1.37 | 1.14 | 1.12 | 1.16 |  |  |  |  |  |
| Consumption of foods rich in vitamin A | Total | - | - | - | - | - | - | - | 65.8 | 67.4 | 67.9 | 64.9 | 63.7 | -2.34 | -7.63 | 3.26 | 0.270 | 0.38 |
|  | q5 | - | - | - | - | - | - | - | 72.4 | 72.0 | 70.9 | 70.1 | 71.36 | -1.98 | -3.62 | -0.32 | **0.036** | 0.93 |
|  | q1 | - | - | - | - | - | - | - | 54.4 | 55.1 | 58.7 | 54.4 | 53.06 | -1.44 | -10.14 | 8.10 | 0.652 | 0.97 |
| **Ratio Q5/Q1** |  | - | - | - | - | - | - | - | 1.33 | 1.31 | 1.21 | 1.29 | 1.34 |  |  |  |  |  |
| Zero consumption of fruit and vegetables  (6 – 23 months) | Total | 14.3 | 12.4 | 10.5 | 12.4 | 10.1 | 11.1 | 8.7 | 8.9 | 8.2 | 7.2 | 8.24 | 9.4 | -10.53 | -14.33 | -6.56 | **<0.001** | 0.68 |
|  | q5 | 4.3 | 7.3 | 6.5 | 6.1 | 5.6 | 5.1 | 6.9 | 5.6 | 5.4 | 5.4 | 5.2 | 5.95 | -1.73 | -6.45 | 3.24 | 0.449 | 0.78 |
|  | q1 | 22.1 | 19.6 | 18.6 | 24.6 | 22.7 | 19.5 | 12.8 | 15.6 | 15.6 | 12.6 | 14.2 | 14.87 | -10.27 | -16.62 | -3.45 | **0.008** | 0.71 |
| **Ratio Q5/Q1** |  | 0.19 | 0.37 | 0.35 | 0.25 | 0.25 | 0.26 | 0.54 | 0.36 | 0.35 | 0.43 | 0.37 | 0.40 |  |  |  |  |  |

APC: annual percentage change. 95% CI: 95% confidence interval. R^2^: coefficient of determination.
